# Identification and characterization of pseudogenes in the rice gene complement

**DOI:** 10.1186/1471-2164-10-317

**Published:** 2009-07-16

**Authors:** Françoise Thibaud-Nissen, Shu Ouyang, C Robin Buell

**Affiliations:** 1 The J. Craig Venter Institute, 9712 Medical Center Dr, Rockville, MD 20850 USA; 2 Current address: National Center for Biotechnology Information, National Institutes of Health, 9000 Rockville Pike, Bethesda MD 20892 USA; 3 Current address: Suite 205, 1003 W. 7th Street, Frederick, MD 21701 USA; 4 Department of Plant Biology, Michigan State University, East Lansing, MI 48824 USA

## Abstract

**Background:**

The Osa1 Genome Annotation of rice (*Oryza sativa *L. ssp. *japonica *cv. Nipponbare) is the product of a semi-automated pipeline that does not explicitly predict pseudogenes. As such, it is likely to mis-annotate pseudogenes as functional genes. A total of 22,033 gene models within the Osa1 Release 5 were investigated as potential pseudogenes as these genes exhibit at least one feature potentially indicative of pseudogenes: lack of transcript support, short coding region, long untranslated region, or, for genes residing within a segmentally duplicated region, lack of a paralog or significantly shorter corresponding paralog.

**Results:**

A total of 1,439 pseudogenes, identified among genes with pseudogene features, were characterized by similarity to fully-supported gene models and the presence of frameshifts or premature translational stop codons. Significant difference in the length of duplicated genes within segmentally-duplicated regions was the optimal indicator of pseudogenization. Among the 816 pseudogenes for which a probable origin could be determined, 75% originated from gene duplication events while 25% were the result of retrotransposition events. A total of 12% of the pseudogenes were expressed. Finally, F-box proteins, BTB/POZ proteins, terpene synthases, chalcone synthases and cytochrome P450 protein families were found to harbor large numbers of pseudogenes.

**Conclusion:**

These pseudogenes still have a detectable open reading frame and are thus distinct from pseudogenes detected within intergenic regions which typically lack definable open reading frames. Families containing the highest number of pseudogenes are fast-evolving families involved in ubiquitination and secondary metabolism.

## Background

Pseudogenes are defined as genes that have lost their ability to produce a functional protein. Although such relics have been identified in all genomes, the number and persistence of pseudogenes varies greatly among species: in human, the estimated number of pseudogenes ranges from 10,000 to 20,000 [1,2], while in Drosophila, only 110 pseudogenes (or 1 pseudogene per 130 genes) were identified [3]. Pseudogenes are hypothesized to arise by gene duplication, including retrotransposition during which a retrotransposase mediates the integration a transcript into the genome [4] (see Additional file 1). Since they are redundant with the genes from which the transcript originated (hereafter termed parent gene) and are integrated without a promoter into random locations in the genome, the products of retrotransposition events are likely to be nonfunctional and to accumulate disabling mutations faster than functional genes. In such cases, they are termed retrotransposed pseudogenes or processed pseudogenes. In general, acceleration of evolutionary rates have been measured immediately following duplication and used to explain functional diversification such as subfunctionalization, neofunctionalization and pseudogenization [5,6].

Limited effort has been put into whole-genome identification of pseudogenes in plants, and, although whole-genome, segmental and tandem duplications have played a large role in the evolution of plant genomes [7,8], most of the literature has focused on the more readily identifiable retrotransposed pseudogenes. The *Arabidopsis *Information Resource (TAIR) has released the annotation of 859 pseudogenes in TAIR8, which were presumably the result of a manual annotation effort [9]. Studies in rice (*Oryza sativa *ssp *indica*) and *Arabidopsis *have focused on chimeric genes originating from the recruitment of additional exons by retrotransposed genes. As by-products of these analyses, Wang et al. [10] found 337 retrotransposed genes containing at least one frameshift mutation in rice, and Zhang et al. [11] reported 22 in *Arabidopsis*. A separate effort using more liberal criteria identified 411 retrotransposed genes in *Arabidopsis*, 376 of which were disabled due to frameshifts or premature stop codons [12].

The majority of studies on pseudogenes focus on the identification of gene relics in the intergenic regions and not among annotated protein coding genes. This is sufficient for highly curated genomes in which pseudogenes have already been annotated. However, an increasing number of genomes are annotated in an automated or semiautomated fashion, and rely partially on *ab initio *gene finders, which typically do not predict pseudogenes. The Osa1 Genome Annotation (of *Oryza sativa *ssp. *japonica *cv. Nipponbare) consists of gene predictions made by the *ab initio *gene finder FGENESH, and improved through incorporation of transcript evidence [13]. Despite expression datasets in the form of Expressed Sequence Tags (ESTs), full-length cDNAs and Massively Parallel Signature Sequencing tags (MPSS), Serial Analysis of Gene Expression (SAGE), and proteomic datasets, over 40% of the non-transposable element (non-TE)-related rice genes are not currently supported by transcript evidence. The *ab initio *gene-prediction software FGENESH was chosen for rice due to its combination of high sensitivity (78%) and specificity (76%) at the exon level [14]. Despite this high performance, FGENESH is likely to circumvent premature stop codons or frameshift mutations leading to premature stop codons in otherwise long open-reading frames (ORF) by adding introns or interrupting the ORF prematurely. Therefore, not only does FGENESH not predict pseudogenes, but it may predict an interrupted ORF where a pseudogene is more likely. Rice pseudogenes annotated by experts and deposited to the Osa1 Community Annotation project are evidence of this issue. Comparison of 72 pseudogenes annotated by community annotators in the Osa1 Release 4 gene annotation revealed that these pseudogenes had either been entirely "missed" by the Osa1 automated pipeline (30 pseudogenes), or had been misannotated (incorrect structures were invoked to circumvent stop codons or frameshifts; 25 pseudogenes), or had been annotated as genes (17 pseudogenes) [15]. These results suggest that a whole-genome approach to the identification of pseudogenes in the rice gene complement would improve the quality of the annotation.

Pseudogene detection methods rely on the alignment of genes to intergenic regions for the identification of a pseudogene-parent pair. The characteristics of the pseudogenes are further determined based on global alignment of the pseudogenes to their respective parents [16-18]. The success of this type of approach is inherently dependent on the quality of the annotation for the organism in question, as it assumes that the structure of the parent gene is accurately predicted [2]. Yao et al. [19] used a different strategy: human genes and pseudogenes were identified by ranking the alignments of EST, mRNA, and protein based on identity and coverage. Models created exclusively from non top-ranking alignments (i.e. non-cognate evidence) were labeled as non-transcribed pseudogenes, while models with cognate transcript(s) but frameshifted cognate protein were designated as transcribed pseudogenes. This approach produced a set of pseudogenes with 75 to 80% overlap with manually curated pseudogenes. An important advantage of this strategy is that it obviates the need for a pre-determined set of functional models. However, the authors also demonstrate that, in the case of the human genome (~20,000 genes), a minimum of 5 million ESTs is necessary to avoid over-predicting pseudogenes, a number vastly superior to what is currently available for rice.

We blended the two methods described above by using only fully-supported rice models to identify pseudogenes among a set of rice genes with features potentially indicative of pseudogenes, hereafter termed Genes with Pseudogene Features (GPFs) (see Additional file 2). Pseudogene features assessed were i) lack of alignment to an EST or cDNA (possibly indicating lack of expression), ii) long untranslated regions (UTRs), iii) short coding sequences (CDS), iv) a downstream poly-A tail, and v) for genes in segmentally-duplicated regions: differing protein length or number of exons between the duplicated genes, or lack of paralog and single-exon gene model structure. Parent-derived models were constructed by aligning all fully-supported gene models (i.e., gene models with full-length cDNA transcript support) to the genomic sequence of GPFs. A total of 1,439 pseudogenes, aligning over at least 70% of the parent and containing disablement(s) (frameshifts and/or premature stop codon) were identified in the rice gene complement. We characterized the pseudogenes, identified their most likely origin, investigated their ancestral function, and validated our method by comparing our results to previously identified pseudogenes in rice.

## Results

### Selection of a set of Genes with Pseudogene Features (GPFs) for further study

In order to avoid over predicting pseudogenes and thereby discarding the annotation of genuinely functional genes, the Osa1 Release 5 gene set was partitioned using criteria that differentiated high-confidence, well-supported genes from lower-confidence functional genes that may be pseudogenes and should be examined. The first criterion was transcript support, as evidence in other organisms suggest that the vast majority of pseudogenes are not transcribed [20]. Reasons for the lack of expression of pseudogenes include the absence of a promoter in the case of retrotransposed pseudogenes and the accumulation of mutations within the promoter of a gene that has been made redundant by another type of duplication event. Among the 41,046 Osa1 Release 5 non-TE-related genes, we identified 17,792 genes without cognate EST or cDNA support (unsupported category, see Table 1) and 831 genes with long 5' or 3' UTRs (long UTR category, Table 1), which could indicate a truncated ORF. We also identified 475 genes with long downstream stretches of adenines, which may be remnants of poly-A tails of transcripts integrated in the genome by a retrotransposase (polyA tail category, Table 1). An additional 734 genes, which were not part of the official Osa1 Release 5 because of the short length of their coding sequence (below 50 amino acids) were also selected for further study (short CDS category, Table 1).

**Table 1:** Genes with pseudogene features (GPFs) and pseudogenes

Category	No. of GPFs	Pseudogenes (%)^1^	Transcribed pseudogenes
Unsupported^2^	17792	1191 (7%)	101 (8.5%)
Long UTR^3^	831	104 (12%)	35 (34%)
Short CDS^4^	734	5(4%)	0 (0%)
Poly-A tail^5^	475	30(6%)	1 (3%)
Segmentally duplicated^6^	248	40(16%)	14 (35%)
Single-exon singletons^7^	4833	202(4%)	31 (15%)
			
Total (non redundant)	22033	1439(6.5%)	170 (13%)

^1 ^Pseudogenes (with parent gene and at least one frameshift or premature stop codon)

^2 ^GPFs not supported by cDNA or EST evidence

^3 ^The UTRs of the GPFs are longer than mean + 2 standard deviations

^4 ^The CDS of the GPFs are shorter than 50 amino acids

^5 ^The GPFs contain a stretch of 18 adenines in a 20-base window, within -200 to 400 bases from the end of the annotated UTR, or within 600 bases of the stop codon if no UTR is annotated

^6 ^The CDS of the GPFs are significantly shorter than their respective paralog or, the GPFs have a significantly smaller number of exons

^7 ^The GPFs contain a single exon and are within a segmentally duplicated region but have no paralog in the duplicated region

To identify additional pseudogenes, we examined genes within segmentally duplicated regions [21]. Among these, 4,833 single-exon genes lacked a corresponding gene in the duplicated segment (single-exon singleton category, Table 1) and could be retrotransposed pseudogenes which inserted after the segmental duplication event. Lastly, we searched for pairs of paralogous genes within duplicated segments [21] that showed a disparity in gene length or exon number between their two members. A total of 248 gene pairs contained a shortened paralog based on CDS length or exon number (segmentally duplicated category, Table 1). In total, 22,033 genes in Osa1 Release 5, hereafter referred to as GPFs, had at least one feature associated with pseudogenes and were selected for further investigation.

### Identification of pseudogenes and parent genes

A total of 5,340 gene models with ≥ 70% coverage of the protein encoded by the parent gene were identified using the strategy summarized in Additional file 2. Among these, 1,439 contained at least one disablement (frameshift or stop codon) and are hereafter termed pseudogenes (Table 1). Only one pseudogene had all disablements in the last 10 amino acid of its sequence (marked with a star in Additional file 3).

Pairwise alignments of the GPFs and the pseudogenes revealed that 75% overlapped, i.e., aligned over > 35 aa with 80% identity or with E-value < 1e-30, indicating that most pseudogenes are variants of the FGENESH model from which the GPFs were derived. This also suggests that the pseudogenes identified in this study may have been recently acquired, and may have diverged less from functional genes than pseudogenes identified within intergenic regions where *ab initio *gene finders are unable to construct a model.

The vast majority of pseudogenes (1,191) originated from the largest group of candidates, the unsupported category. Beyond the absolute numbers, the percentage of pseudogenes identified from the GPFs in each category varied from 0.7% to 16% (Table 1). Significant differences in size within segmentally duplicated genes and unusually long UTRs were the best indicators of pseudogenization, with 40 (16%) and 104 (12%) of the GPFs in these categories respectively identified as pseudogenes. A short CDS and singleton status within a segmentally duplicated region were the least robust predictors for pseudogenization, with 5 (<1%) and 202 (4%) pseudogenes, respectively. It should be noted that the percentage of pseudogenes identified in each category depends in part on the identification of a parent for the candidate pseudogene. Any pseudogene that has diverged from its parent gene (<40% identity), or which has lost over 30% of its coding region, would not be identified within the parameters used in this study.

### Duplicated pseudogenes are more abundant than retrotransposed pseudogenes

Number of exons within the pseudogene and corresponding parent gene was used to determine the pseudogenization mechanism. Retrotransposed pseudogenes are expected to be single-exon genes regardless of the structure of the parent, while duplicated pseudogenes to have retained at least some of the ancestral introns based on the low rates of intron gain and loss observed in rice [21]. We were able to derive the pseudogenization mechanism for the 816 (57%) pseudogenes with multi-exon parents (Table 2). Of these, 77% were multi-exon pseudogenes (i.e., duplicated pseudogenes) while 23% were single-exon pseudogenes (i.e., retrotransposed pseudogenes). Among the remaining 43% pseudogenes (all with single-exon parents and therefore unresolved origin) an overwhelming majority are single-exon pseudogenes with single-exon parents (86%, 539 out of 623), and a small proportion consist of multi-exon pseudogenes predicted to originate from a single-exon parent (13%, 84 out of 623). It is possible that the introns in this last group of pseudogenes are mis-predicted or appeared after the retrotransposition or duplication event, and originate from the insertion of a retroelement [17,18].

**Table 2:** Origin of the pseudogenes

	Known		Unknown	
Category*	Duplicated	Retrotransposed	Single-exon pseudogene	Multi-exon pseudogne
Unsupported	507	162	453	69
Long UTR	62	11	25	6
Short CDS	1	0	4	0
Poly-A tail	9	2	16	3
Segmentally duplicated	36	2	1	1
Single-exon singletons	39	34	115	14
				
Total (non redundant)	627	189	539	84

*See Table 1 for a description of the different categories

The pseudogenes were evenly distributed throughout the genome (see Additional file 4). Examination of the distributions of retrotransposed and duplicated pseudogenes, segmentally duplicated regions and tandemly duplicated genes suggest that pseudogenes are not disproportionately associated with segmentally duplicated regions or clusters of tandemly replicated genes (Additional file 4).

As expected, almost all of the pseudogenes identified in paralogous pairs within segmentally duplicated regions are of duplicated origin (36 out of 38). Among the pseudogenes of known origin, a significantly higher proportion of retrotransposed pseudogenes were identified in the single-exon singleton category (34 out of 73 of known origin or 46%, versus 189 out of 816 or 23% across categories, p-value < 10^-5^, Fisher's exact test), thereby verifying our hypothesis that many of these pseudogenes might have appeared by retrotransposition subsequent to the major segmental duplication event that occurred in rice 70 million years ago [22].

### Characteristics of the pseudogenes

Comparison of the characteristics in duplicated versus retrotransposed pseudogenes (Table 3) indicates that pseudogenes of duplicated origin are on average longer (492 versus 398 amino acids) and more similar to their respective parent genes as measured by nucleotide identity, protein similarity, and percent coverage. The number of disablements is slightly higher in duplicated compared to retrotransposed pseudogenes (1.85 versus 1.52), but this trend is reversed when the number of disablements is normalized by the length of pseudogenes, as expressed in the number of disablements per 1000 bases. These statistics suggest that retrotransposed pseudogenes have diverged more from their parent gene than duplicated pseudogenes.

**Table 3:** Characteristics of the pseudogenes

	Length (aa)	Nucleotide identity (%)	Protein similarity (%)	Coverage (%)	Disablements/pseudogene	Disablements/1000 bases
Duplicated	492	70.3	73.3	89.9	1.85	5.03
Retrotransposed	398	59.2	63.8	85.8	1.52	5.69
Unknown single-exon						
pseudogene	257	71.6	70.8	91	1.83	10.32
Unknown, multi-exon						
pseudogene	455	63.0	63.7	89.7	1.96	6.91

Table 3 also shows that pseudogenes of unknown origin with single-exon parents have characteristics that are more similar to those of duplicated pseudogenes compared to those of retrotransposed origin (with the exception of their shorter length). This suggests that the majority of these pseudogenes may have been generated by duplication.

**Figure 1:**
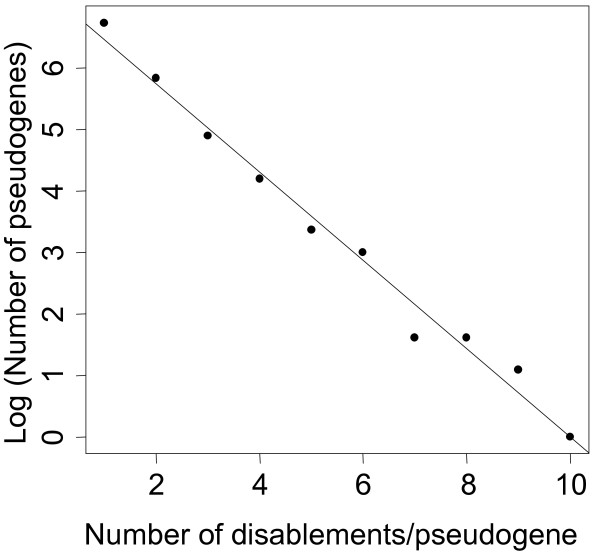
**Number of disablements per pseudogene**. The number of disablements is represented on the x-axis and the log normal of the number of pseudogenes on the y-axis.

The distribution of the number of disablements per pseudogene was plotted in Figure 1. Retrotransposed, duplicated, and genes of unknown origin all followed the same relationship (not shown). The distinctive exponential relationship between the number of disablements and the number of pseudogenes suggests that the appearance of disablements in pseudogenes is random and may be corollary to reduced selective pressure on the pseudogenes [23].

### Expression of the pseudogenes

Several reports of a small but significant proportion of expressed pseudogenes in the human genome [2,20,24] prompted us to look at the expression level of pseudogenes in rice. Given the fact that 83% of the pseudogenes identified are in the unsupported category as defined by lack of EST and full-length cDNA support (Table 1), we investigated deeper expression evidence datasets provided by MPSS expression profiles. We searched for MPSS tags identified in 22 rice libraries [25] that mapped uniquely to pseudogene exons. Overall, 170 pseudogenes (12% of the total) showed at least some basal expression in the MPSS libraries surveyed, compared to 844 parent genes (92% of the total number of parent genes). However, the level of expression of these pseudogenes was significantly lower than that of their respective parent (163 versus 486 transcripts per million, *p *= 0.03, pairwise t-test), which is consistent with the lack of EST and/or full-length cDNA support for the majority of the associated GPFs. The proportion of transcribed pseudogenes ranged from 0% in the short CDS category to 35% in the duplicate category (Table 1). Altogether, 133 (78%) of the transcribed pseudogenes were of known origin: among them, 114 (85%) were of duplicated origin and 19 (15%) were retrotransposed. Based on the total number of duplicated and retrotransposed pseudogenes (627 and 189 respectively), these results indicate that 18% of the duplicated pseudogenes are transcribed versus only 10% of the retrotransposed pseudogenes. This difference is consistent with observations in human [2] and is likely due to the fact that integration of mRNA by a retrotransposase is random and does not necessarily occur proximal to a promoter.

### Rate of non-synonymous to synonymous substitution of pseudogenes

**Figure 2:**
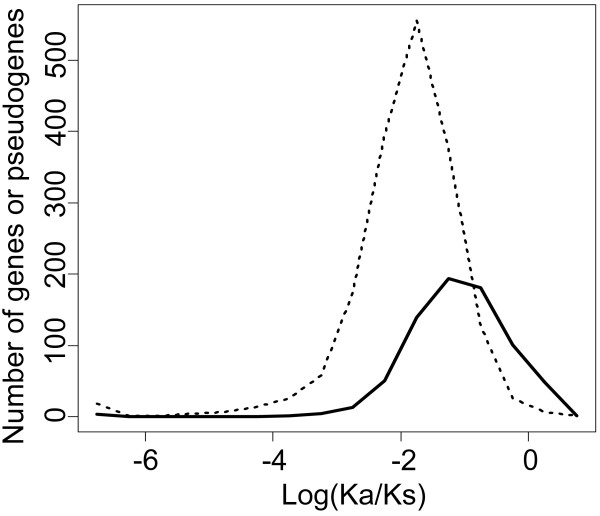
**log(Ka/Ks) ratios distribution of the pseudogenes (full line) and of a control set of functional paralogous genes (dotted line)**.

Due to their non-functional nature, pseudogenes are not expected to be under evolutionary constraint and instead, are expected to be under neutral selection. Thus, pseudogenes should have a synonymous substitution rate (Ks) roughly equal to the non-synonymous mutation rate (Ka), while functional genes should have a Ka/Ks much lower than 1, since non-synonymous mutations present a disadvantage and are selected against (purifying selection). Maximum likelihood estimates of the Ka and Ks were calculated by analysis of the alignments of the pseudogenes to their corresponding parents. We found that the Ka/Ks distribution was log-normal with a geometric mean of 0.32. This is lower than the expected value of 1, but can be explained in part by the fact that each pseudogene is compared to its "sibling" rather than its true, ancestral, parent. This approximation inflates the Ks value and therefore decreases the Ka/Ks. Furthermore, we estimated the Ka/Ks for paralogous functional genes within segmentally duplicated regions [21] in the same manner as for the pseudogenes and compared the Ka/Ks distribution of the pseudogenes to that of this control set (geometric mean of 0.14). The two distributions were found to be significantly different (*p *< 10^-15^, Welch t-test) (Figure 2).

### Evaluation of pseudogene detection method with manually curated pseudogenes

**Figure 3:**
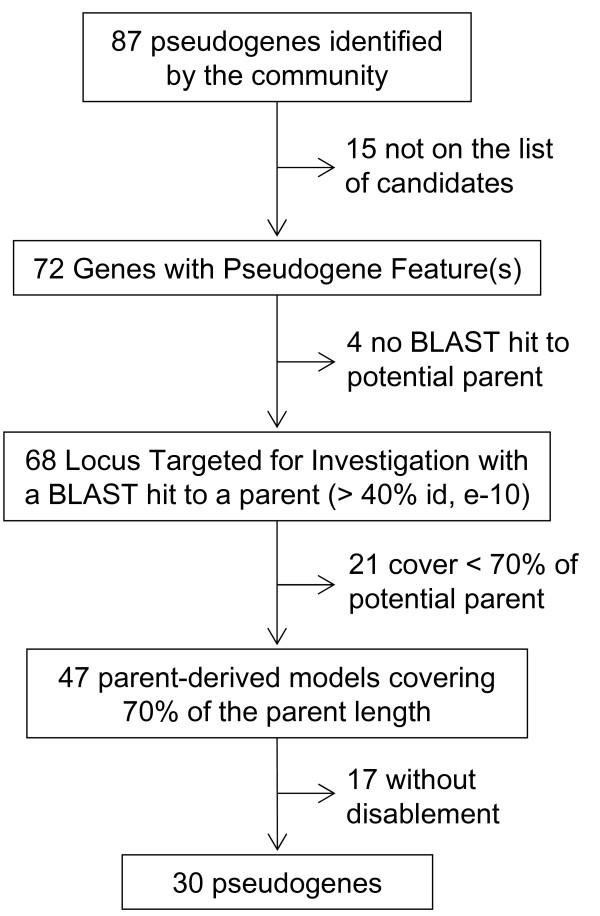
**Fate of the community-annotated pseudogenes in our annotation process**. Number of candidates passing each step in our pseudogene identification method.

Because they are defined by their non-functional nature, pseudogenes can not be verified experimentally. In the absence of a set of pseudogenes defined with certainty, we used a set of pseudogenes manually curated by the community to benchmark our pseudogene identification pipeline. As of December 2007, over 2,200 rice genes have been annotated by experts and deposited within the Osa1 Community Annotation project [15]. For some families (Bric-a-Brac/Tramtrack/Broad Complex proteins (BTB/POZ), wall-associated kinases (WAKs), cysteine-rich peptides, glycosyl hydrolase family 1, HKT sodium and potassium transporters), annotation of pseudogenes as well as of functional genes was provided. These community annotated models were obtained by querying the six-frame translation of the rice genome with well-characterized proteins or sequence motifs and were subjected to manual curation [26-30]. A total of 87 annotated pseudogenes in these families overlap with Osa1 Release 5 gene models while other genes are predicted within intergenic regions of the genome. We found that 30 of the 87 community-annotated pseudogenes were accurately predicted as pseudogenes in our pipeline. More importantly, 72 of the 87 (83%) community-annotated pseudogenes were GPFs. This proportion is significantly higher than the proportion of Osa1 Release 5 gene models selected as GPFs (53%, *p *< 10^-8^, Fisher's exact test). As seen in Figure 3, a BLAST alignment was obtained for the majority of the GPFs identified as pseudogenes by the community. However, many pseudogenes identified by the community annotators did not pass the strict coverage and disablement criteria set in our pipeline and as a consequence, were not annotated as pseudogenes in the present study.

### Ancestral function of the pseudogenes

The ancestral function of each pseudogene was determined based on the Gene Ontology Slim terms associated with the pseudogene's corresponding parent, since the pseudogenes may have undergone sequence loss, and since, in the extant genome, the parent gene best represents the ancestral gene from which the pseudogene originated. A total of 513 parents corresponding to 687 pseudogenes were found to be associated with one or more GO terms [13]. Comparison of the relative frequency of each GO term in the overall Osa1 gene complement versus the pseudogenes revealed an over-representation of the genes involved in secondary metabolism, amino acid and derivative metabolic process, signal transduction, and kinase activity (Table 4).

**Table 4:** Twenty most significantly over-represented GO terms in pseudogenes

GO term	Number of pseudogenes	Percent of pseudogenes	Percent of Osa1 Gene Complement	p-value	GO term description
GO:0019748	250	36.4	11.7	2.5E-63	Secondary metabolic process
GO:0009058	277	40.3	16.9	1.1E-47	Biosynthetic process
GO:0008150	186	27.1	9.6	3.8E-39	Biological process
GO:0006519	162	23.6	9.0	3.0E-30	Amino acid and derivative metabolic process
GO:0007165	341	49.6	29.0	3.5E-30	Signal transduction
GO:0016301	418	60.8	41.7	2.3E-24	Kinase activity
GO:0005739	248	36.1	19.6	4.0E-24	Mitochondrion
GO:0030246	101	14.7	5.2	7.0E-21	Carbohydrate binding
GO:0009987	254	37.0	21.5	7.1E-21	Cellular process
GO:0016740	216	31.4	17.7	7.9E-19	Transferase activity
GO:0007582	228	33.2	19.5	1.2E-17	Physiological process
GO:0009719	300	43.7	29.3	5.8E-16	Response to endogenous stimulus
GO:0016020	280	40.8	26.8	8.2E-16	Membrane
GO:0006629	103	15.0	6.5	3.2E-15	Lipid metabolic process
GO:0005515	189	27.5	16.3	6.1E-14	Protein binding
GO:0005618	165	24.0	13.9	5.6E-13	Cell wall
GO:0004872	51	7.4	2.5	8.7E-12	Receptor activity
GO:0030154	57	8.3	3.2	5.2E-11	Cell differentiation
GO:0005886	73	10.6	4.7	1.3E-10	Plasma membrane
GO:0006464	138	20.1	11.8	1.8E-10	Protein modification process

**Figure 4:**
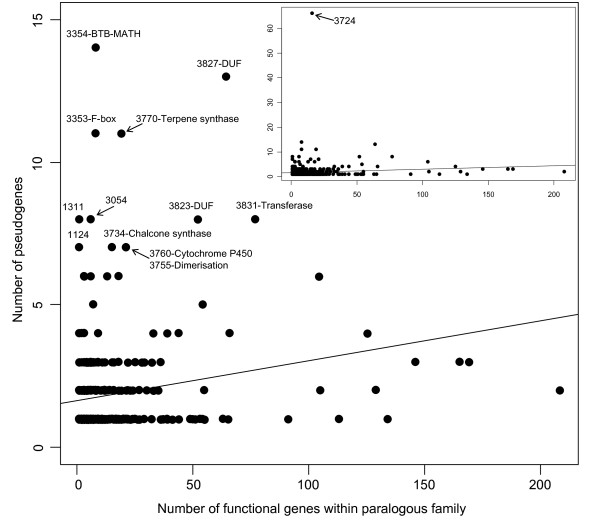
**Number of pseudogenes per paralogous family**. Pseudogenes were associated with paralogous families, based on their parents. Families discussed in the text are labeled with their number and the name of the associated Pfam domain, if characterized. BTB-MATH: Bric-a-Brac/Tramtrack/Broad Complex and Meprin and TRAF homology domain. DUF: domain of unknown function. The straight line represents the linear regression of the number of pseudogenes per family over the number functional genes per family. In the inserted plot, the y-axis has a greater range to represent family 3724.

In order to refine this general categorization, the pseudogenization frequency was examined within paralogous families that were constructed through clustering of PFAM and novel domains of the entire rice proteome [31]. A total of 558 parents of 815 pseudogenes belonging to 444 paralogous families were examined. The number of pseudogenes per family was plotted against the size of the family (Figure 4). The scatter of the data suggests that the number of pseudogenes per paralogous family is poorly correlated to the size of the family (*r*^2 ^= 0.01).

Three families involved in ubiquitination contained a notable number of pseudogenes. Family 3354, which contains 14 pseudogenes and 8 functional genes are characterized by a MATH (Meprin and TRAF homology (PF00917)) and a BTB/POZ (PF00651) domains. Two families, 3353 and 3352, containing F-box domains (PF00646) have respectively 8 pseudogenes for 11 functional genes and 6 pseudogenes for 6 functional genes. Both F-box and BTB/POZ proteins assure the function of substrate recognition during ubiquitination [26,32].

Most of the other families with a large proportion of pseudogenes are involved in secondary metabolism and have transferase activity, consistent with the over-representation of these two terms in our GOSlim analyses. Family 3734 containing the chalcone/stilbene synthase domain PF00195, the chalcone/stilbene synthase C-terminal domain PF02797, and the 3-oxoacyl [acyl-carrier-protein] synthase III C terminal domain PF08541 comprises 15 functional genes and 7 pseudogenes. Chalcone synthases catalyze the first committed step in the flavonoid pathway, which produces a wide range of secondary compounds. Family 3755 (21 functional genes) is characterized by the dimerisation domain PF08100 and the 6 parent genes of the 7 pseudogenes in this family are annotated as O-methyltransferases with homology to maize ZRP4, an enzyme of the phenylpropanoid pathway involved in the production of suberin [33]. Family 3770 comprises 11 pseudogenes and 27 genes characterized by the metal binding domain PF03936 and the N-terminal domain PF01397 of terpene synthases, a family of enzymes catalyzing the first step in many pathways leading to a wide range of secondary compounds and to gibberellic acid. Family 3760 (21 functional genes and 7 pseudogenes) contains the cytochrome P450 domain PF00067. Cytochrome P450s play an important role in hormone synthesis (gibberellic acid, abscissic acid and brassinosteroids) and in secondary metabolism. These pseudogenes contributed largely to the enrichment of the GO term GO:0006519 (amino acid and derivative metabolic process) in our GOSlim analysis.

In addition, several families with no known domain or with domain of unknown function were found to be enriched in pseudogenes such as families 1311, 1124 and 3054 (Figure 4). Most strikingly, the paralogous family 3724, which contains 19 functional genes, was found to have accumulated 66 pseudogenes, the largest number for any given family. These single-exon pseudogenes are children of 3 single-exon parents, with no identified PFAM domains, and one uncharacterized domain identified by sequence homology.

## Discussion

### Number of pseudogenes in the rice gene complement

A total of 1,439 pseudogenes were identified among the ~41,800 non-TE-related genes annotated in Osa1 Release 5. Altogether, the presence of retrotransposed or duplicated pseudogene characteristics was investigated in a subset of the non-TE-related genes (22,033, 53%). To our knowledge, our study is the first attempt at identifying and characterizing pseudogenes of duplicated origin in a plant species. While we identified 1,439 pseudogenes in this study, these represent only a partial set of pseudogenes in the rice genome as we deliberately designed a conservative approach to annotating pseudogenes to prevent mis-annotation of true functional genes. First, we limited our analysis to a set of genes that are weakly supported by transcript evidence and/or exhibit features of pseudogenes, thereby limiting the number of functional genes examined. Second, although disablements can be considered to be a consequence of the loss of functionality of a gene rather than a cause, and are therefore, by some definition, not a required feature of pseudogenes [17], we required the presence of frameshift(s) or a premature stop codon in our pseudogene set. It should be mentioned that only a minute number of pseudogenes are likely to be the product of a sequencing errors, which was estimated at 1 in 10,000 bases in the finished rice genome sequence [34]. Third, only fully-supported high-confidence models were used as potential parents for the pseudogenes to limit the propagation of errors from the parent to the pseudogene [2]. This implies that pseudogenes with poorly expressed parents may not be identified. Fourth, identity and coverage thresholds used for the alignment of the parent to the candidate pseudogene regions were conservative, although within range of what had been used in similar analyses [1,17].

### Retrotransposed versus duplicated pseudogenes

Assignment of a probable mechanism of origination was possible for over half of the pseudogenes based on the internal structure of the parent gene and pseudogene. Pseudogenes of duplicated origin are more abundant than pseudogenes of retrotransposed origin across all categories that were considered (overall ratio of 3 to 1). Moreover, comparison of duplicated and retrotransposed pseudogene alignments with their corresponding parent gene suggests that pseudogenes of unknown origin are likely to have arisen by duplication. This high ratio of duplicated versus retrotransposed pseudogenes differs from observations in human where retrotransposition is the source of 70–75% of the identified pseudogenes [2,18] and in which the appearance of pseudogenes has been linked to a burst in L1 retrotransposon activity 40–50 million years ago [35]. However, the duplicated to retrotransposed pseudogene ratio is consistent with the important role of duplication in the shaping of the rice genome. By some estimates, over 50% of the genome could be the product of duplication [7,8].

Alignments of pseudogenes to their parents showed that retrotransposed pseudogenes are more diverged from their parent gene than their duplicated counterparts. This observation is consistent with the fact that products of retrotransposition, in the absence of a nearby promoter, are, in essence, pseudogenes as soon as they are inserted in the genome ("dead-on-arrival" [36]), and begin accumulating mutations faster than duplicated genes which remain functional for a period of time after duplication. Therefore, the prevalence of pseudogenes of duplicated origin might be accentuated by the fact that a portion of retrotransposed pseudogenes are too degenerated to be identified by our method, and we can not discard the possibility that retrotransposed pseudogenes are more abundant in the intergenic regions.

### Pseudogenes are most abundant in fast-evolving gene families involved in ubiquitination and secondary metabolism

Several large rice families, such as the BTB/POZ or the cytochrome P450 family are known to contain a large proportion of pseudogenes [26,37]. Gingerich *et al*.[26] identified 149 functional genes and 43 pseudogenes encoding BTB proteins in rice, 20 of which were also identified by our method. At least 99 pseudogenes for 328 functional cytochrome P450s were identified in rice [37], and on a smaller scale Itoh *et al*. [38] identified a pseudogene in a cluster of rice ent-kaurene oxidase genes. Although, to our knowledge, no terpene synthase or chalcone/stilbene synthase pseudogene has been reported in rice, a whole-genome survey of terpene synthases in *Arabidopsis *identified a core of 32 closely related terpene synthases and 8 pseudogenes [39]. There has also been reports of pseudogenes in the chalcone synthase family of *Ipomoea *[40], in the Asteraceae genus *Dendranthema *[41] and in *Trifolium subterraneum *[42]. The fact that results obtained through our automated pipeline are consistent with manual annotation is additional evidence of the genuine nature of the pseudogenes in our set.

Despite the fact that superfamilies such as the cytochrome P450 or F-box proteins contain a high number of pseudogenes, the correlation between the number of pseudogenes per family and the size of the family was found to be low (Figure 4). This apparent contradiction can be explained by the high granularity of the set of paralogous families used here. Proteins were separated into paralogous families based not only on PFAM domains but also on uncharacterized domains identified through protein alignments [31]. The low correlation between number of pseudogenes and family size suggests that within a large family, the pseudogenes are often circumscribed to a subfamily of proteins. A notable exception is the pseudogenes associated with kinases. Based on GO term analysis, a kinase ancestral function can be attributed to 418 pseudogenes (60% of these with a GO term, Table 4). However, these pseudogenes are distributed among a large number of paralogous families characterized with a kinase domain. As a consequence, none of these families was found to contain a noticeably large number of pseudogenes.

The families containing a large number of pseudogenes share functional and evolutionary characteristics. Collectively, terpene synthases catalyze the first committed step to the several pathways producing primary compounds such as gibberellins, carrotenoids as well as pathways that produce a wide range of secondary compounds, many of them expressed in response to pathogen attack [43]. Some members of the cytochrome P450 family are involved in the synthesis of gibberrellins, abscissic acid, brassinosteroids and many take part in the synthesis of phenylpropanoids (phytoalexins) [44]. Chalcone/stilbene synthases are the gate-keepers of the flavonoid biosynthetic pathway, which lead to the synthesis of the anthocyanins responsible for flower color as well as a variety of compounds with a role in plant pathogen interactions [45]. The BTB proteins are part of the BTB E3 ligase complex and are responsible for the recognition of the targets to be ubiquitinated, a role similar to that of the F-box proteins in the SCF (Skp1p-cullin-F-box) E3 ligase complex [46]. Therefore, many families rich in pseudogenes participate in the synthesis of defense compounds or in the recognition of molecules destined for degradation.

In addition, these families contain phylogenetic clusters of lineage-specific genes. Such indication of recent expansion has been shown for the BTB/MATH branch of the BTB proteins in rice, the branch that harbors the 20 BTB pseudogenes that were identified in this study and Gingerich *et al*. [26]. Similar observations have been made for the F-box proteins in rice [32,47]. Phylogenetic analyses have shown that terpene synthases are more similar within than across species, indicating that many functions have evolved repeatedly in different species. The same is true of the chalcone synthase family, which has been the subject of tandem duplication in multiple species [40,48].

Finally, enzymatic plasticity has been reported for the terpene synthases and the chalcone synthases. Substitution of a few amino acids in the catalytic site of chalcone synthase turns the enzyme into a stilbene synthase [48]. In the terpene synthase family, a single amino acid difference observed in the catalytic sites of two orthologs of kaurene synthase in *indica *and *japonica *rice shifts the product outcome from *ent*-isokaur-15-ene, an intermediate in the synthesis of gibberellin to the secondary compound ent-pimara-8(14),15-diene [49]. Changes *in vitro *of a few amino acids in the catalytic site of a diterpene synthase from Norway spruce radically changes the reaction outcome from a single product (isopimaradiene) to several (abietadiene, levopimaradiene, neoabietadiene and palustradiene) [50].

## Conclusion

We have identified 1,439 pseudogenes in the rice gene complement for which an ORF is still detectable. A large number of these pseudogenes are members of fast-evolving families in plants and have a role in the response to biotic stresses and in ubiquitination. As plants adapt to a changing environment and evolution of pathogens, expanded subfamilies of genes involved in plant defense may act as sandboxes from which some genes emerge as advantageous and are subjected to positive selection while some are not and become pseudogenes.

## Methods

### Selection of genes targeted for investigation and parent genes

Parent genes and GPFs were identified within the Osa1 Release 5 gene complement [13]. All TE-related genes were removed from the Osa1 Release 5 gene set and, in the event of alternative splice forms, only the representative gene model (with the longest coding region) was used. This set of 41,046 genes was augmented with 734 genes with CDS shorter than 50 amino-acids [51]. In total, 41,780 genes were used in this study.

The parent gene set (16,284 genes) was defined as genes fully supported by ESTs or full-length cDNAs [13]. GPFs were defined as: i) genes with no full-length cDNA or EST support as specified in the feature file provided on the Osa1 FTP site [51], ii) genes predicted to encode proteins of less than 50 amino acids, iii) genes with 5' or/and 3' UTRs over 2 standard deviations (SD) above the geometric mean UTR length as calculated on the log normal distribution of the UTR length (1,155 nt for 5' and 1,408 nt for 3' UTR), or iv) 1- to 2-exon genes with the remnant of a poly-A tail defined as at least 17 adenines in a stretch of 20 bases located between -200 and +1400 bp of the gene's translational stop codon if the gene has no reported 3' UTR, or between -200 and 400 bp of the 3' end of the gene if the gene has a poly-A tail. These large windows were based on the calculation of the mean + 2SD of 3' UTRs and took into account that, for many genes, the extent of the UTR has not been defined, and that the program used in gene model construction tends to over-predict the length of UTRs [9].

In addition, GPFs were also selected in segmentally duplicated regions by examining pairs of non-TE related paralogous genes [21]. The mean and SD of the difference in exon number in the coding region between duplicated genes were calculated to be 0 and 1.98, respectively. Mean and standard deviation of the difference in the protein length between the two members in each pair were calculated to be 0 and 137 amino acids, respectively. Pairs, for which the absolute difference in length or exon number was above 2*SD were selected for further analysis with the longest gene in the pair hypothesized to be the parent gene, and the shortest the gene targeted for investigation. Finally, non-TE single-exon genes located in segmentally duplicated regions that lacked a duplicate gene were targeted for investigation.

### BLAST searches

With the exception of the genes in the segmentally duplicated category which, by definition, have a pre-determined parent, parent genes were identified by alignment of the 16,284 fully-supported genes annotated in Osa1 Release 5 to the genomic sequence of the GPFs, hereafter referred to as Locus Targeted for Investigation (LTIs, see Additional file 2). A LTI was defined as the genomic sequence of a GPF with a buffer of 100 bases flanking the GPF (see Additional file 2). The parent gene set was searched, using TBLASTN, against all the LTIs (with the exception of short genes in segmentally duplicated regions for which the long paralog is the parent) with E value < 10^-10 ^and identity cut-off ≥ 40% [17]. The BLAST results were parsed using a set of perl scripts to identify the best non-overlapping aligning protein(s) to each candidate region. Similar to PseudoPipe [16], the alignments of a single protein to a LTI were "merged" into super-alignments by recording the left-most and right-most coordinates of all the alignments for the subject-query pair. Overlapping and redundant super-alignments from different proteins were then resolved by selecting the multi-exon protein comprising the alignment with the smallest E-value as the putative parent gene for that sub-region. In this manner, a LTI can be paired with more than one group of non-overlapping alignments which could lead to several parent genes and hence several pseudogenes [16]. Multi-exon genes with less homology to the LTI were given precedence over single-exon genes due to the possibility that single-exon gene parent might themselves be of retrotransposed origin [10]. In cases where no alignment was derived from multi-exon genes, the protein with the smallest E value was selected as the parent.

### Global alignment of loci targeted for investigation to parent genes

The coordinates of the LTI were recalculated so that the alignment determined by BLAST was at the center and flanked on each side by a genomic region three times the size of the putative parent protein. This adjustment permitted more optimal global alignment of the putative parent in instances when the latter aligned only partially and to the extremity of the candidate region in the BLAST step.

The global alignment tool GeneWise [52] was used to determine the best parent-derived model that could be constructed in the LTI by aligning the parent gene to that region. GeneWise was chosen due to its allowance of stop codons and frameshifts in the predicted model, and therefore its ability to predict putative pseudogenes. Parent-derived models covering at least 70% of their respective parent protein and containing at least one disablement (frameshift or premature stop codon) were termed pseudogenes (see Additional file 2). The pseudogene proteins and nucleotide sequences, number of exons in the coding region of the parent proteins and pseudogenes, number of frameshifts and stop codons in the pseudogenes, length of the pseudogenes and parent proteins were derived from the GeneWise output.

### Substitution rate ratio in the pseudogenes

Parent genes and pseudogenes were aligned using CLUSTALW [53] with the default parameters. A maximum likelihood estimate of the synonymous substitution rate Ks (*d*_*S*_, number of synonymous substitution per synonymous site) and the nonsynonymous substitution rate Ka (*d*_*N*_, number of nonsynonymous substitution per nonsynonymous site) was calculated using the PAML 3.15 *codeml *package, running in pairwise mode (runmode = -2), with the equilibrium codon frequencies calculated from the average nucleotide frequencies at the three codon positions (CodonFreq = 2) [54]. The Ka/Ks ratios of paralogous genes in segmentally duplicated regions [21] which were not candidate pseudogenes were calculated in the same manner. The difference in the distribution of the log(Ka/Ks) in the control and the pseudogene set was estimated using a Welch two-sample t-test with unequal variance as implemented in the R function *t. test*. Only alignments longer than 100 amino acids and with non-saturated Ks (Ks < 2) were used in the analysis.

### Pseudogene expression

Expression of the pseudogenes was inferred from MPSS data from 22 libraries [25] using previous mapping of 17 and 20-bp MPSS tags to the rice genome [13]. A gene or pseudogene was annotated as transcribed when at least one MPSS tag mapped uniquely and entirely to an exon. Average count per million for each tag was calculated as the sum of the counts per million in each library, as provided by the Rice MPSS database [25]. In cases where several tags mapped to a gene, the tag with the maximum frequency was selected to represent the expression of the gene.

### Pseudogene function

GOSlim terms were assigned to Osa1 Release 5 genes based on sequence similarity to *Arabidopsis *genes as described previously [13]. Each pseudogene was attributed the GOSlim term(s) of its corresponding parent gene, since the parent gene is the closest representation of the ancestral gene from which the pseudogene arose. Relative frequencies of each GOSlim term in the Osa1 Release 5 gene set versus the pseudogene set were calculated and over-representation of GOSlim terms was determined based on the Fisher's exact test, as implemented in the R *fisher. test *function. Only genes with at least one GOSlim term were taken into consideration in the calculation.

In order to obtain a more granular view of the pseudogenes' ancestral function, we examined the distribution of the pseudogenes in paralogous families, as classified by Lin *et al*. [31]. As in the GOSlim analysis, each pseudogene was assigned to the paralogous family of its parent. All GPFs for which a pseudogene was identified were removed from the paralogous families, so that only one gene or pseudogene per locus was counted. For each family, a count of the numbers of genes and pseudogenes was obtained.

## Authors' contributions

FT and CRB conceived the study and wrote the manuscript, FT identified the candidate sets and the pseudogenes, SO performed the GOSlim anotations and mapped the pseudogenes to the genome. All authors read and approved the final manuscript.

## List of abbreviations

TAIR: The *Arabidopsis *Information Resource; TE: transposable element; ORF: open-reading frames; GPF: Gene with Pseudogene Features; UTR: untranslated region; CDS: short coding sequence; MPSS: Massively Parallel Signature Sequencing; LTI: Locus Targeted for Investigation; SD: standard deviation;

## Supplementary Material

Additional data file 1 **Two possible origins for pseudogenes**. This figure shows two possible mechanisms by which pseudogenes originate. A. duplication, B. retrotransposition. The colored blocks represent exons, the lines introns or intergenic regions. The thick vertical red lines represent frameshifts or premature stop codons. Click here for file

Additional data file 2 **Definitions used in this article**. Genes with Pseudogene Features (GPF, in blue) were identified first. The corresponding loci, with flanking buffer regions were termed Locus Targeted for Investigation (LTI, thick black lines). Parent genes were identified by searching fully-supported genes against the LTIs. The parent-derived models were created by re-aligning each parent gene to the corresponding LTI with GeneWise. Pseudogenes were defined as parent-derived models with disablements (thick red vertical lines) and covering at least 70% of the parent protein. Click here for file

Additional data file 3 **List and attributes of the 1,439 pseudogenes identified in the rice gene complement**. A list of the 1,429 pseudogenes identified in the rice gene complement along with their attributes. Click here for file

Additional data file 4 **Distribution of the pseudogenes in the rice genome**. Distribution of the pseudogenes in the rice genome. Purple vertical bars: pseudogenes of unknown origin, dark green vertical bars: retrotransposed pseudogenes, red vertical bars: duplicated pseudogenes, blue vertical bars: tandemly duplicated genes, green segments: segmental duplication, black segments: centromeres. Click here for file
