# Supplementary material for: Identification and characterization of pseudogenes in the rice gene complement
Source: BMC Genomics. 2009 Jul 16;10:317. doi: 10.1186/1471-2164-10-317 (PMC2724416; doi:10.1186/1471-2164-10-317)
Supplement: Additional data file 1 — Two possible origins for pseudogenes. This figure shows two possible mechanisms by which pseudogenes originate. A. duplication, B. retrotransposition. The colored blocks represent exons, the lines introns or intergenic regions. The thick vertical red lines represent frameshifts or premature stop codons. [file 1471-2164-10-317-S1.pdf]

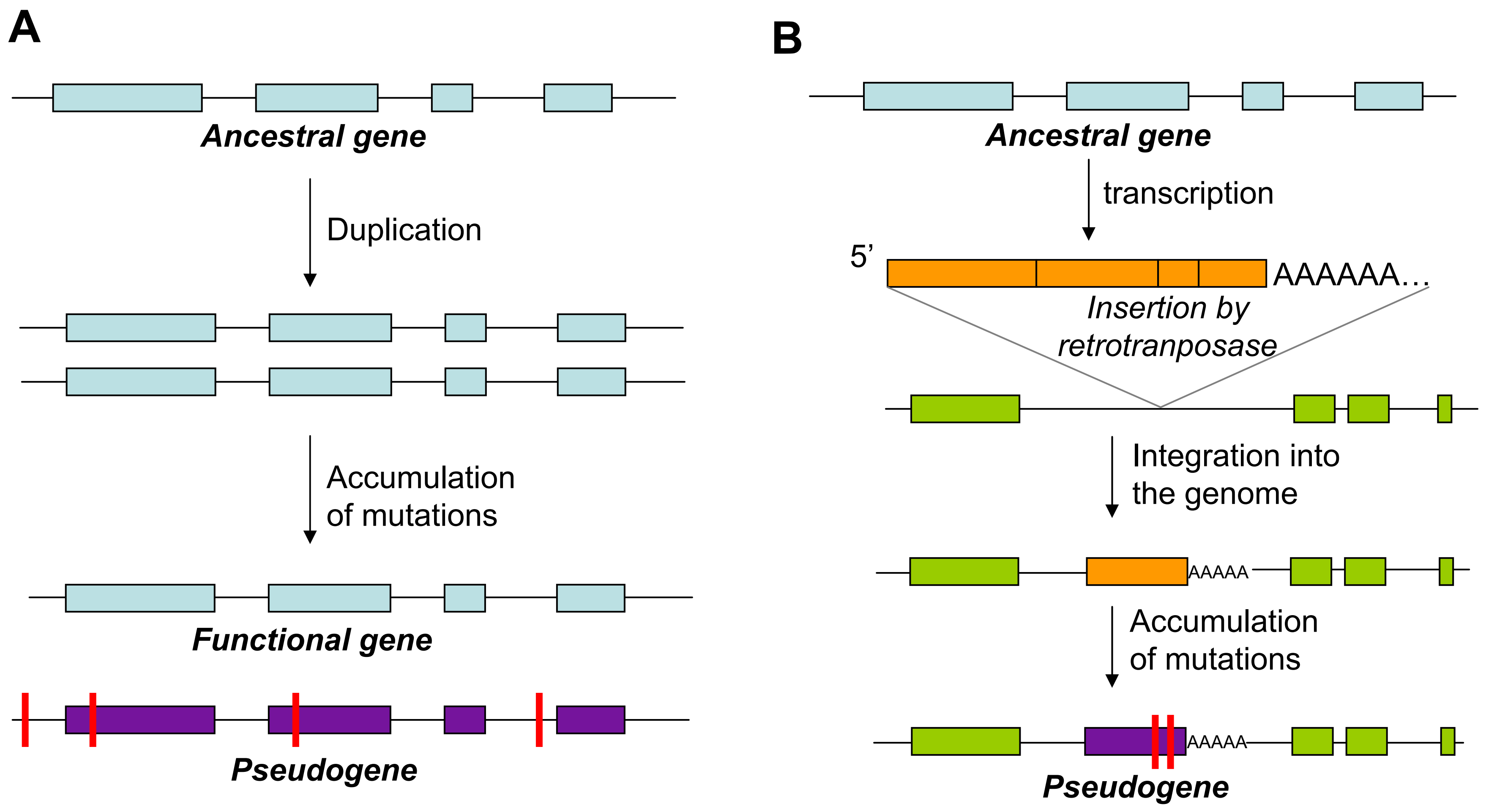

Additional file 1. Two possible origins for pseudogenes

A. duplication, **B.** retrotransposition. The colored blocks represent exons, the lines introns or intergenic regions. The thick vertical red lines represent frameshifts or premature stop codons.
