# Supplementary material for: Identification and characterization of pseudogenes in the rice gene complement
Source: BMC Genomics. 2009 Jul 16;10:317. doi: 10.1186/1471-2164-10-317 (PMC2724416; doi:10.1186/1471-2164-10-317)
Supplement: Additional data file 2 — Definitions used in this article. Genes with Pseudogene Features (GPF, in blue) were identified first. The corresponding loci, with flanking buffer regions were termed Locus Targeted for Investigation (LTI, thick black lines). Parent genes were identified by searching fully-supported genes against the LTIs. The parent-derived models were created by re-aligning each parent gene to the corresponding LTI with GeneWise. Pseudogenes were defined as parent-derived models with disablements (thick red vertical lines) and covering at least 70% of the parent protein. [file 1471-2164-10-317-S2.pdf]

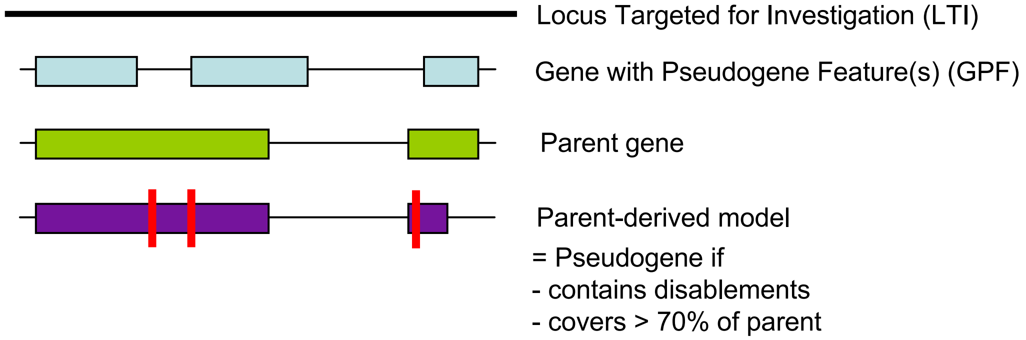

Additional file 2: Definitions

Genes with Pseudogene Features (GPF, in blue) were identified first. The corresponding loci, with flanking buffer regions were termed Locus Targeted for Investigation (LTI, thick black lines). Parent genes were identified by searching fully-supported genes against the LTIs. The parent-derived models were created by re-aligning each parent gene to the corresponding LTI with GeneWise. Pseudogenes were defined as parent-derived models with disablements (thick red vertical lines) and covering at least 70% of the parent protein.
