# Supplementary material for: Identification and characterization of pseudogenes in the rice gene complement
Source: BMC Genomics. 2009 Jul 16;10:317. doi: 10.1186/1471-2164-10-317 (PMC2724416; doi:10.1186/1471-2164-10-317)
Supplement: Additional data file 4 — Distribution of the pseudogenes in the rice genome. Distribution of the pseudogenes in the rice genome. Purple vertical bars: pseudogenes of unknown origin, dark green vertical bars: retrotransposed pseudogenes, red vertical bars: duplicated pseudogenes, blue vertical bars: tandemly duplicated genes, green segments: segmental duplication, black segments: centromeres. [file 1471-2164-10-317-S4.pdf]

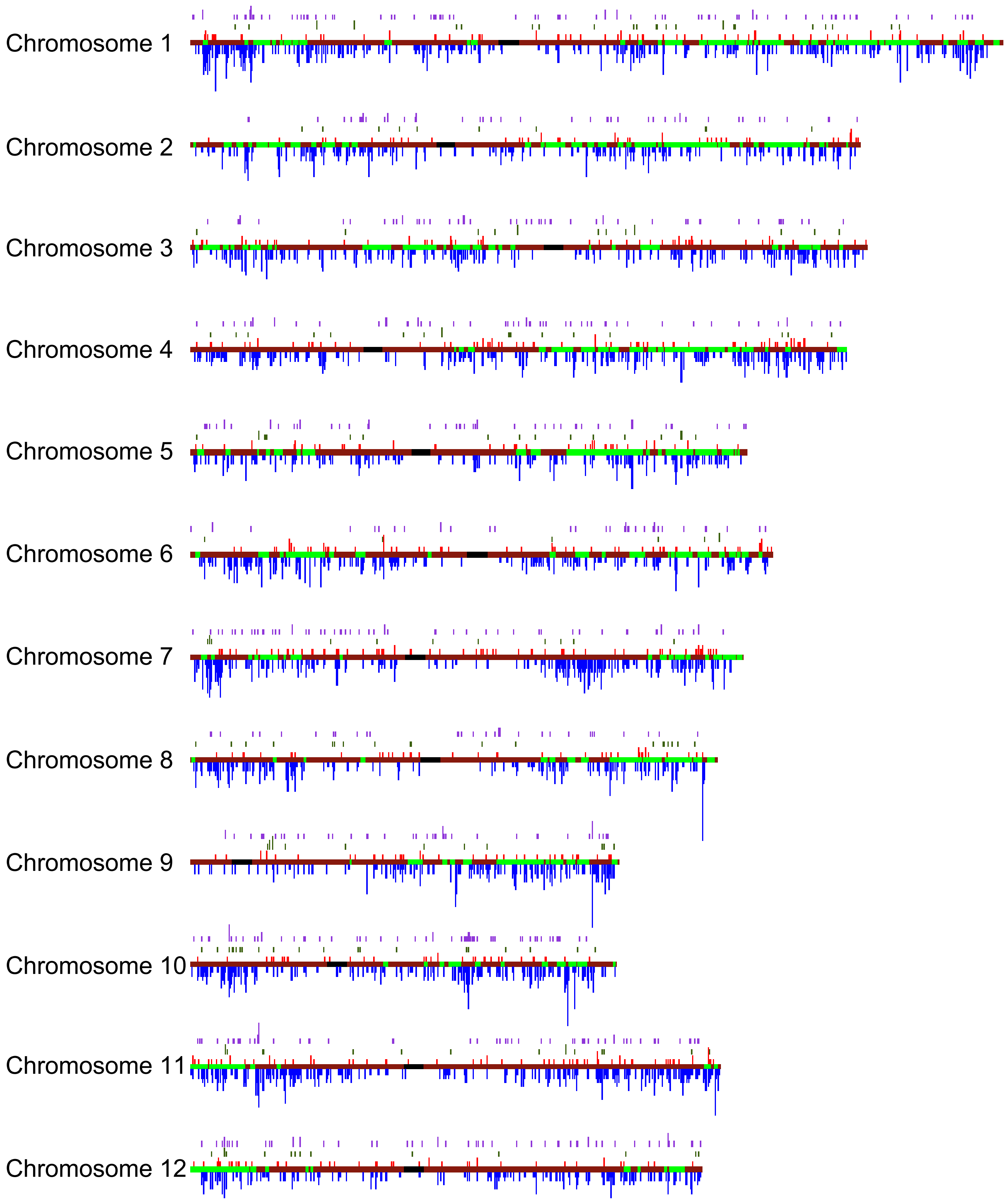

Additional file 4: Distribution of the pseudogenes in the rice genome
Purple vertical bars: pseudogenes of unknown origin, dark green vertical bars: retrotransposed pseudogenes, red vertical bars: duplicated pseudogenes, blue vertical bars: tandemly duplicated genes, green segments: segmental duplication, black segments: centromeres.
